# Neuroinflammation in Aged Brain: Impact of the Oral Administration of Ellagic Acid Microdispersion

**DOI:** 10.3390/ijms21103631

**Published:** 2020-05-21

**Authors:** Raffaella Boggia, Federica Turrini, Alessandra Roggeri, Guendalina Olivero, Francesca Cisani, Tommaso Bonfiglio, Maria Summa, Massimo Grilli, Gabriele Caviglioli, Silvana Alfei, Paola Zunin, Rosalia Bertorelli, Anna Pittaluga

**Affiliations:** 1 Department of Pharmacy (DiFar), University of Genoa, Viale Cembrano 4, 16148 Genoa, Italy; boggia@difar.unige.it (R.B.); turrini@difar.unige.it (F.T.); roggeri@difar.unige.it (A.R.); olivero@difar.unige.it (G.O.); cisani@difar.unige.it (F.C.); grilli@difar.unige.it (M.G.); caviglioli@difar.unige.it (G.C.); alfei@difar.unige.it (S.A.); zunin@difar.unige.it (P.Z.); 2 Department of Internal Medicine, University of Genoa, Viale Benedetto XV 6, 16132 Genoa, Italy; bonfigliot@gmail.com; 3 Translational Pharmacology, Istituto Italiano di Tecnologia, Via Morego 30, 16163 Genoa, Italy; Maria.Summa@iit.it (M.S.); Rosalia.Bertorelli@iit.it (R.B.); 4 Department of Pharmacy (DiFar), Center of Excellence for Biomedical Research, 3Rs Center, University of Genoa, Viale Cembrano 4, 16148 Genoa, Italy; 5 IRCCS Ospedale Policlinico San Martino, 16145 Genova, Italy

**Keywords:** ellagic acid (EA), EA microdispersion (EAm), oral administration, mice, aging, behavioral skills, principal component analysis (PCA), noradrenaline, GFAP, CD45

## Abstract

The immune system and the central nervous system message each other to preserving central homeostasis. Both systems undergo changes during aging that determine central age-related defects. Ellagic acid (EA) is a natural product which is beneficial in both peripheral and central diseases, including aging. We analyzed the impact of the oral administration of a new oral ellagic acid micro-dispersion (EAm), that largely increased the EA solubility, in young and old mice. Oral EAm did not modify animal weight and behavioral skills in young and old mice, but significantly recovered changes in “ex-vivo, in vitro” parameters in old animals. Cortical noradrenaline exocytosis decreased in aged mice. EAm administration did not modify noradrenaline overflow in young animals, but recovered it in old mice. Furthermore, GFAP staining was increased in the cortex of aged mice, while IBA-1 and CD45 immunopositivities were unchanged when compared to young ones. EAm treatment significantly reduced CD45 signal in both young and old cortical lysates; it diminished GFAP immunopositivity in young mice, but failed to affect IBA-1 expression in both young and old animals. Finally, EAm treatment significantly reduced IL1beta expression in old mice. These results suggest that EAm is beneficial to aging and represents a nutraceutical ingredient for elders.

## 1. Introduction

Aging is a progressive physio-pathological process that provokes an array of both central and peripheral complications. The central derangements cause a progressive loss of function leading to neurological symptoms and severe cognitive decline. Learning and memory impairments as well as mood disorders and anxiety mainly rely on alteration of the chemical neurotransmission in selected regions of the central nervous system (CNS), particularly affecting the catecholaminergic, the cholinergic and the GABAergic pathways. These systems are selected targets of aging processes, as the efficiency of the synthesis and the release of these neuromodulators, as well as the expression of the corresponding membrane receptors, result in impairments in old brains [1,2,3,4,5,6].

Aging represents a crucial topic in the management of public health and influences financial health policies worldwide. The growing confidence in the promise of new actions/interventions that could in part restore the neuronal damages and/or delay their progression is so far unanswered and, despite expectations, the need of therapeutics remains unmet.

Among all the promising therapeutic trials, in recent years, scientists’ interests about natural remedies has grown and lead one to suggest nutritional approaches as potential beneficial treatments for anti-aging intervention [7,8,9]. Natural or processed foods can be defined as functional foods if they contain known biologically active compounds (i.e., nutraceuticals), which can provide clinically proven and documented health benefits, including the prevention, management and treatment of chronic diseases [10,11] beyond their nutritional content. Among the functional foods there is the pomegranate fruit [12], that recently gained huge popularity as a nutraceuticals source, becoming a high value crop. Pomegranates are particularly rich in ellagitannins (ETs), which are hydrolysable tannins containing ellagic acid (EA) as common aglycone. The health promise relies on both preclinical and clinical findings, which highlighted the beneficial effects of pomegranates and its main constituents in the treatment of peripheral disorders, such as cardiovascular disease, diabetes, obesity and prostate cancer, but also in central pathologies typified by neuroinflammation and neurodegenerative processes, including multiple sclerosis, Alzheimer’s disease, depression and Parkinson’s disease [13,14,15,16].

The use of pomegranate fruits and/or their main components is limited by the pharmacokinetic profile. In vivo, ETs are rather not absorbed, but preferentially hydrolyzed in the gastrointestinal tract to EA that, again for its trivial water-solubility and first pass effect, has a very low bioavailability [17], and it is here preferentially metabolized to urolithins (UROs), that rapidly diffuse and circulate in plasma as glucuronide and sulfate conjugates to target tissues [18,19].

Boggia and colleagues [20] recently proposed a new formulation that consists of an EA micro-dispersion (EAm), prepared with low methoxylated pectin by spray drying technology, which largely increases the water solubility of EA, from 9.7 μg/mL to 300 μg/mL [17,21]. This formulation would be expected to ameliorate the pharmacokinetic profile of EA itself and could represent a new bioactive ingredient to enrich functional foods or to formulate dietary supplements, and even potentially be investigated as a drug. In preliminary experiments, performed to verify the palatability of the micro-dispersion administered to mice, the new formulation did not limit the access of animals to the beverage. This observation opened the possibility to test the efficacy of the EAm in preclinical studies, as it permits chronic oral treatment, avoiding any form of stress due to forced-feeding by gavage [22].

In a recent study [6], we demonstrated that aging affects the central noradrenergic pathway, significantly reducing noradrenaline exocytosis. This maladaptation could negatively reverberate on the functional cross-talk linking the CNS and the immune system (IS), favoring neuroinflammation and pathological activation of glial cells, that, in turn, may be detrimental to brain functions. The functionality of noradrenergic system also depends on oxidative pathways that finely tune its efficiency. We wondered whether the administration of the EAm could recover the noradrenergic defects, being beneficial in elders. The results of this study support the use of EA for a therapeutic dietary supplementation in elders.

## 2. Results

### 2.1. The Chronic Oral Administration of the EAm in Young and Aged Mice: Control of Daily Intake and Weight Gain

The EAm drug loading (DL) percentage has been spectrophotometrically and chromatographically monitored, and it is equal to 22%, as already described by the authors in a recent paper [20]. The drinking solutions for the animal treatment were prepared daily, dissolving EAm in mineral water (1:1 *w*/*w*), and its EADL % was spectrophotometrically checked within the 24 h. The UV-visible spectra of the 14 days drinking solutions, prepared both for old and young mice respectively within a single treatment, were averaged and pre-treated by standard normal variate (SNV) [23]. SNV is a pretreatment particularly used for spectral data to correct both baseline shifts and global intensity variations of each UV-visible spectrum. The UV-visible spectra were comparable along the 14 days of treatment and they allowed one to calculate the EA DL value percentage of the drinking solutions. The mean DL percentage during the 14 days of treatment was equal to 22.0 + 0.5%).

A preliminary treatment was carried out to assess whether the administration of EAm dissolved in the drinking water could modify the daily access of mice to the beverage. The daily intake (mL) of the EAm suspension in young mice did not differ significantly from the amount of the daily water intake (mL) in control, untreated, animals (untreated young mice: 5.37 ± 0.55 mL; EA-treated mice: 5.54 ± 0.48, *n* = 8, n.s.), consistent with the conclusion that EAm does not significantly modify the palatability of the drinking solution.

**Figure 1:**
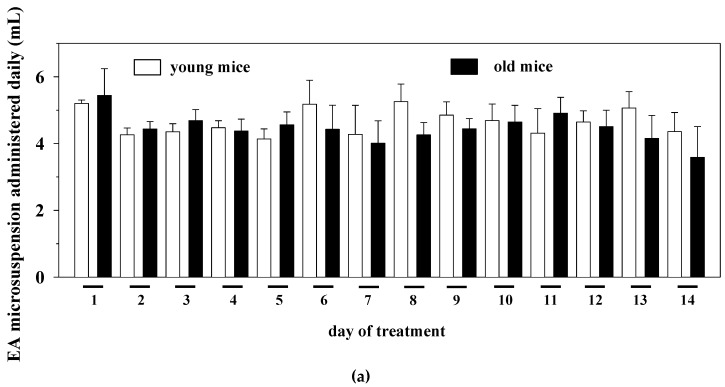

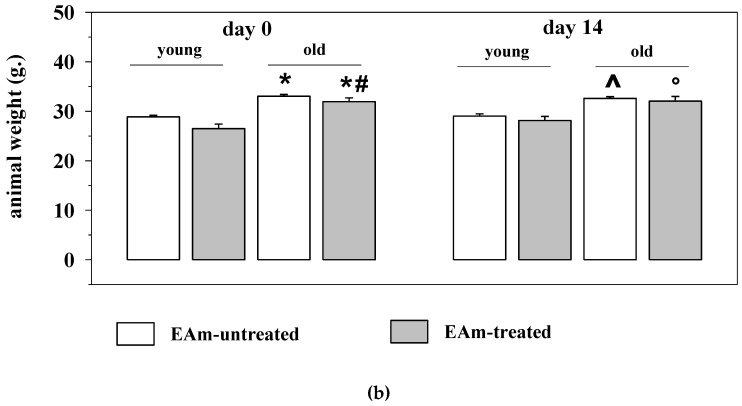
Daily intake and animal weight changes during the ellagic acid micro-dispersion (EAm) treatment. (**a**) daily EAm intake (mL) in young (empty bar) and old (black bar) mice during the 14 days EAm treatment. The daily intake is expressed as mean± SEM of the amount of EAm taken up by 8 animals for each group. (**b**) Animal weight (g.) in EAm-untreated (empty bar) or EAm-treated (gray bar) young and old mice before (day 0) and at the end (day14) of the EAm treatment. The weight is expressed as mean ± SEM of the amount of EAm taken up by *n* = 8 animals for each group. Statistical analysis was performed by applying ANOVA followed by Tukey’s Multiple Comparison test. * *p* < 0.05 vs. young mice at day 0 of EAm-treatment; # *p* < 0.05 vs. old mice at day 0 of EAm-treatment; ^ *p* < 0.05 vs. young mice at day 14 of EAm-treatment; ° *p* < 0.05 vs. old mice at day 14 of EAm-treatment.

Based on these observations, young (three months old) and old (twenty months old) mice were administered chronically (14 days) with the EAm suspension (EA dose: 50 mg/Kg/day). The amount of the microdispersion taken up was monitored daily. During the 14 days’ treatment, the old mice drank a volume of the EAm largely comparable to that taken up by young individuals (Figure 1a). The weight of the mice before and after the 14 days’ EAm treatment was also monitored. The EAm administration did not affect the weight of both young and old mice when compared to respective untreated animals. Nonetheless, the weight of the old untreated mice at the beginning and at the end of the experiment significantly differed from that of the young untreated individuals and, similarly, the weight of the old treated mice at the beginning of the drug treatment significantly differed from that of young treated animals (Figure 1b).

### 2.2. Evaluation of the Effects of the Oral Administration of EAm on Behavioural Skills in “In Vivo” Studies in Young and Aged Mice Using a Univariate Approach

Owing to highlight whether EAm administration could have an impact in young and old mice, EAm untreated and treated animals were analyzed for their behavioral skills in the light-dark maze in two sets of experiments carried out at different times, which involved 35 animals (17 young mice and 18 old ones). Behavioral skills were quantified as the number of transitions from the dark to the light side of the cage (number of transition), as well as the amount of time (expressed as seconds) that the animal spent in the light side of the cage (time in the light). The number of transitions gives a measure of the spontaneous motor activity of the mice, but it also indirectly permits one to unveil their anxious phenotype. An indirect measure of the anxiety of the mice is also obtained by quantifying the time spent by the animals in the light side of the maze. Animal behavioral skills were analyzed before (day 0), during (day 7) and at the end (day 14) of the drug treatment.

**Figure 2:**
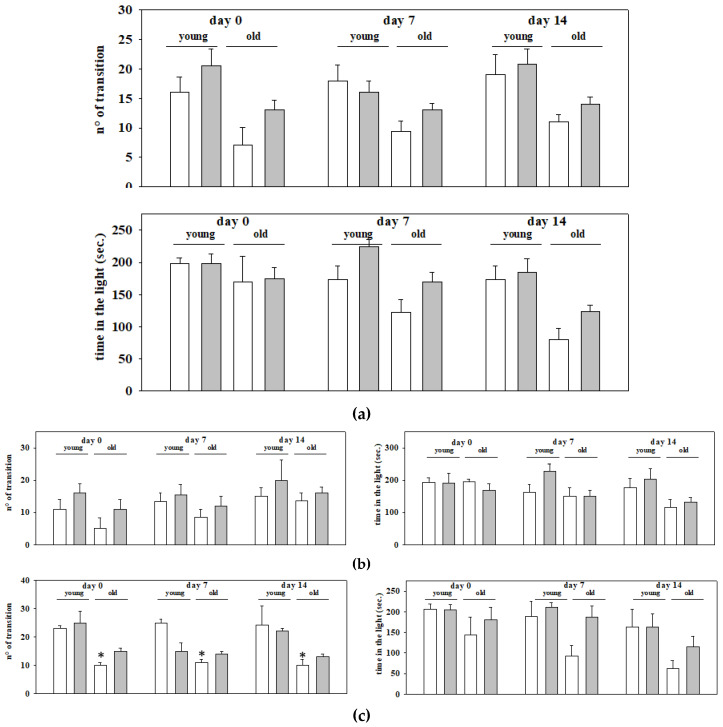
Motor and anxiety behavior in young and old mice: effects of chronic EAm treatment. EAm-untreated (empty bar) or EAm-treated (gray bar) young and old mice were analyzed for explorative and anxiety-related behavior in the light dark box. Behavioral skills were quantified as number of transitions between the two sides of the maze (n° transitions), as well as the total time spent in the light compartment of the light dark box (time in the light), before (Day 0), during (day 7) and at the end (day14) of the EAm administration. (**a**) Data represent the mean ± S.E.M. of 35 animals (17 young mice and 18 old ones) analyzed in two different trials carried out on different days. (**b**) Data represent the mean ± S.E.M. of the 17 animals (9 young mice and 8 old ones) analyzed in the first trial; (**c**) Data represent the mean ± S.E.M. of the 18 animals (8 young mice and 10 old ones) analyzed in the second trial. Statistical analysis was performed by applying ANOVA followed by the Tukey’s Multiple Comparison test. * *p* < 0.05 vs. EAm-untreated young mice.

The analysis with the univariate descriptive statistical approach of the results from the two sets of experiments did not highlight significant differences between the young and the old mice (Figure 2a). The analysis also unveiled that EAm treated animals (young and old ones as well) did not differ significantly from the respective controls (Figure 2a). Comparable conclusions emerged when analyzing the results from each of the two sets of experiments (Figure 2b,c), with the exception of the results from the second set, which unveiled a significant difference in the time spent in the light side by the old untreated mice when compared to the young untreated ones (Figure 2c).

### 2.3. Evaluation of the Effects of the Oral Administration of EAm on Behavioural Skills in “In Vivo” Studies in Young and Aged Mice Using a Multivariate Approach

Even if univariate methods are helpful in extracting useful information from data, it is, however, widely recognized that they offer a limited overview of the results as a whole, since they consider one variable at a time, independently of the others, leading one to underestimate the potential differences among groups within the same test.

The limit can be overcome by applying multivariate statistical methods that allow one to extract the global information present in a dataset, considering inter-correlation among variables, providing a holistic interpretation of the data structure. Based on this assumption, but also owing to analyze whether the data so far available could give in a whole some information on the EAm oral administration, a principal component analysis (PCA) [24,25] was performed.

PCA is a mathematical tool able to study the data structure, look for similarities or dissimilarities among objects (e.g., to highlight possible differences between young and old mice that could depend on the EAm administration) and highlight outliers (if present) in the multivariate datasets under study. It is probably the simplest and the most widely used method in the explorative multivariate analysis to rationalize the useful information of a dataset.

In order to analyze behavioral skills data by PCA, a merged dataset using the data coming from the two behavioral trials have been prepared. The 35 animals (dataset objects) have been divided into the following four categories: untreated young mice (CTR_YOUNG), untreated old mice (CTR_OLD), EAm-treated young mice (EA_YOUNG), EAm-treated old mice (EA_OLD), each described by the variables (dataset columns) coming from the behavioral test results plus the animal weight variation. Thus, PCA was performed on the merged data matrix, namely T_35,7_, whose 35 rows represent the animals of the two sessions, and the columns are the following seven variables: the results of the behavioral tests performed respectively at day 0, day 7 and day 14, plus the animal weight variation variable (DW: delta weight).

Autoscaling, the most common pre-processing method in multivariate analysis, which consists of mean-centering followed by the division of each column (variable) by its standard deviation, has been applied before PCA, to elaborate the multivariate data characterized by different scales and units.

Subsequently, PCA was analogously performed on the two treatments separately organizing the corresponding data in the following two data matrices, namely F_17,7_ and S_18,7_, for the first and the second treatment, respectively. 

**Figure 3:**
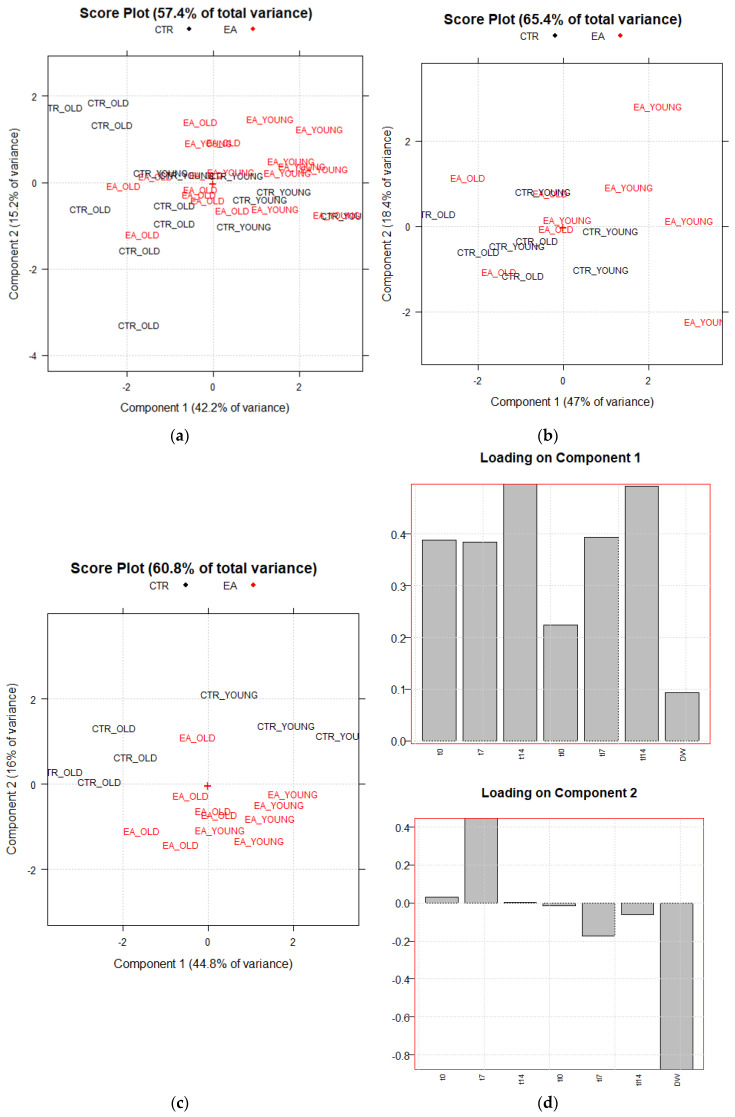
Principal Component Analysis results. PC1 vs. PC2 Score plots of PCA performed respectively on T_35,7_ and F_17,7_ data matrices (**a**,**b**). Score and Loading plots of PCA performed on S_18,7_ data matrix (**c**,**d**). CTR_YOUNG: untreated young mice; CTR_OLD: untreated old mice; EA_YOUNG: EAm-treated young mice; EA_OLD: EAm-treated old mice.

PCA plots (score and loading plots) show the interrelations existing among samples, among variables, and between samples and variables. Figure 3a–c shows the PCA results obtained from the abovementioned three matrices (T_35,7_; F_17,7_; S_18,7_). The first score plot (Figure 3a) obtained from the PCA on T_35,7_ is poorly informative, since no significant separation among the four groups of animals is highlighted when all the data from the two behavioral trials are elaborated together. Nevertheless, Figure 3b, obtained just from the first trial, shows that most of the EAm-treated young mice have more positive scores on the first PC than the untreated young mice. A trend is thus highlighted, even if just for young animals. This trend is more evident in the second trial (matrix: S_18,7_, Figure 3c). The scores plot on PC1–PC2 (Figure 3c), which visualizes 60.8% of the total explained variance corresponding to the 60.8% of the total information of the dataset, highlights a separation among the above cited four categories of animals. Two big groups: old (black ink) vs. young (red ink) animals are discriminated on PC1, that is the direction of maximum variance mainly built by t14 and tl14 variables (highest loadings on PC1, see Figure 3d), whose values are much higher in young compared to old animals. Nevertheless, more interestingly, together with PC1, PC2 also contributes to separate untreated vs. EAm-treated animals. The EAm-treated old animals have scores more like EAm-treated young mice respect to untreated animals, presenting similar values of both t7 and of DW, with the highest loadings on PC2 (Figure 3d).

### 2.4. Effects of the Oral Administration of EAm on “Ex Vivo, In Vitro” Cortical Noradrenergic Synaptosomes from Young and Aged Mice

Based on the results from the multivariate analysis, we asked whether EAm administration could have caused some functional adaptations in the CNS of both young and aged mice, insufficient to cause a clear-cut modification of the behavioral skills in “in vivo” studies, but sufficient to prime cellular events controlling synaptic plasticity and central resilience.

Data exist in the literature concerning the impact of aging on central chemical transmission and some of them agree with the main role that catecholaminergic (i.e., the dopaminergic and the noradrenergic) impairments exert on the onset of functional age-related central defects [2]. In a recent paper, we demonstrated that the exocytosis of noradrenaline from cortical nerve endings is significantly reduced in aged mice when compared to controls [6]. Because of the main role of this transmitter in the control of anxiety and mood, experiments were dedicated to analyzing the impact of EAm administration on the release and the uptake of the catecholamine in young and old mice.

**Figure 4:**
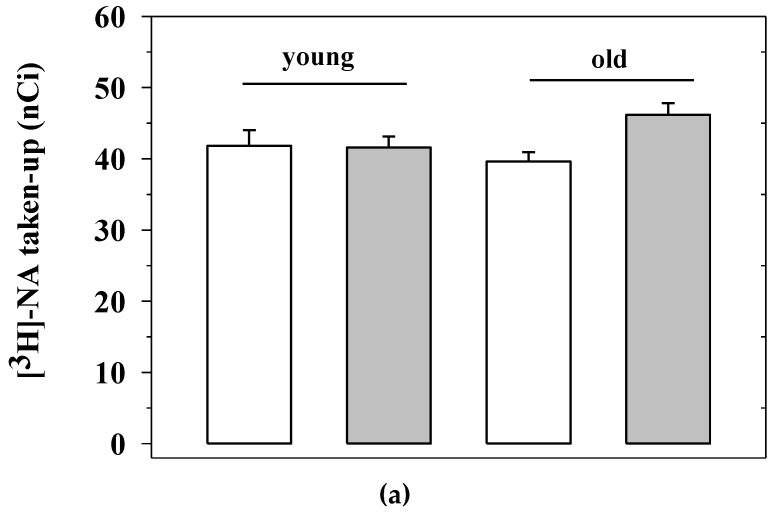

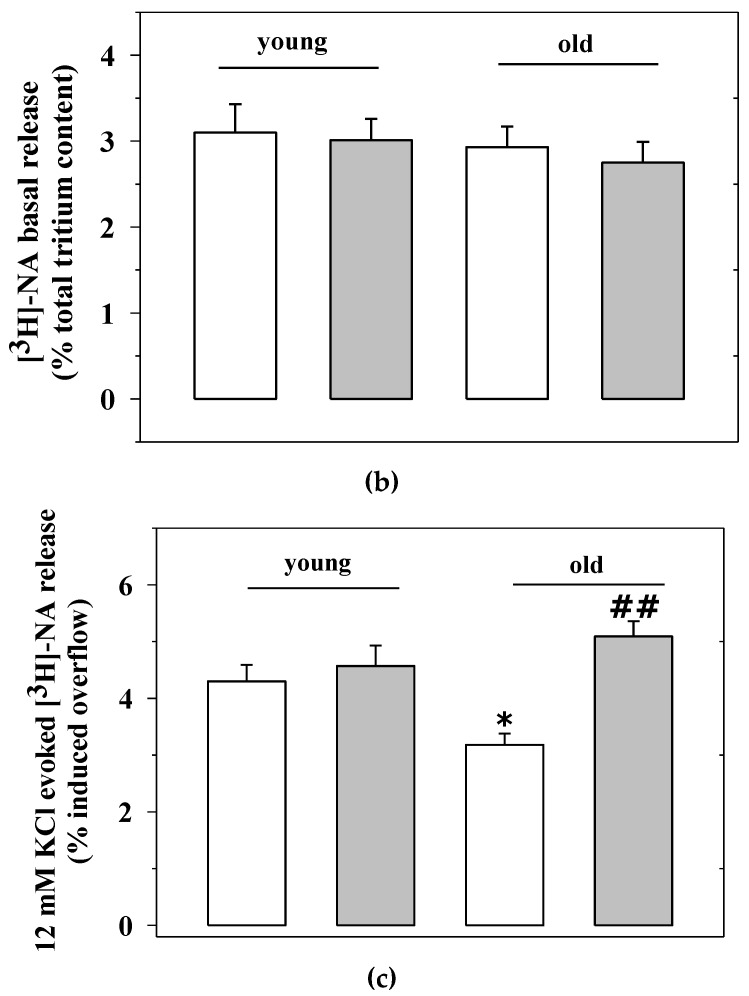
Noradrenaline uptake and release from cortical synaptosomes of young and old mice: effects of chronic EAm treatment. (**a**) total [^3^H]noradrenaline ([^3^H]NA) content (expressed as nCi) in cortical synaptosomes from EAm-untreated (empty bar) or EAm-treated (gray bar) young and old mice. (**b**) spontaneous basal release (expressed as percentage of tritium in the first collected superfused fraction, with respect to the total tritium synaptosomal content) in cortical synaptosomes from the four animal groups. (**c**) 12 mM KCl-evoked [^3^H]noradrenaline release (expressed as percentage of tritium released over the basal outflow with respect to the total tritium synaptosomal content) in cortical synaptosomes from the four animal groups. Data represent the mean± S.E.M. from nine untreated young mice, eight EAm-treated young mice, nine untreated old mice, nine EAm-treated old mice, from both of the two trials. Statistical analysis was performed by applying ANOVA followed by the Tukey’s Multiple Comparison test. * *p* < 0.05 vs. EAm-untreated young mice; ## *p* < 0.01 vs. EAm-untreated old mice.

Briefly, synaptosomes isolated from the mice of the four experimental animal groups analyzed for behavioral skills (i.e., the untreated young mice, the EAm-treated young mice, the untreated old mice, the EAm-treated old mice) were labelled with [^3^H]noradrenaline ([[^3^H]NA), to monitor the efficiency of NA uptake and release. The NA uptake efficiency in the cortical synaptosomes of aged mice did not significantly differ from that detected in young animals, nor did the EAm administration significantly influence this parameter in either young or old animals (Figure 4a).

Cortical synaptosomes labelled with [^3^H]NA were then monitored to quantify both the basal and the depolarization-evoked release of tritium [5,26]. The basal release of [^3^H]NA (i.e., the amount of tritium released in the 3 min fraction collected immediately before that containing the depolarization-evoked release of amine) from cortical synaptosomes of aged mice was comparable to that from cortical terminals from young animals (well consistent with previous data [6]). The “in vivo” administration of EAm failed to modify this “ex vivo, in vitro” parameter in both young and aged animals (Figure 4b).

The “in vivo” administration of EAm, however, significantly affected the [^3^H]NA exocytosis from the cortical synaptosomes of aged animals. The transient (90 s) exposure of cortical synaptosomes to a mild depolarizing stimulus (i.e., 12 mM KCl) elicits the Ca^2+^-dependent, exocytotic-like release of [^3^H]NA. The results from the release studies confirmed our previous observations, showing that the 12 mM KCl-evoked exocytosis of noradrenaline from cortical synaptosomes of aged mice is significantly lower than that observed from cortical synaptosomes of young animals (Figure 4c). The “in vivo” administration of EAm did not alter the release of preloaded [^3^H]NA from cortical synaptosomes from young mice, but significantly increased the 12 mM KCl-evoked tritium overflow from cortical synaptosomes from aged mice, to a level that was largely comparable to that observed in young mice (Figure 4c).

### 2.5. Effects of the Oral Administration of EAm on Markers that Typify Inflammation in the Cortex of Young and Aged Mice

During aging, central neurological defects are often associated with increased basal inflammation, typified by microglia activation, astrocytosis and lymphocytes invasion [27,28,29].

Based on the alterations of the cytokine levels observed in old untreated and treated mice, experiments were first dedicated to quantifying the expression of CD45 (here used as s marker of lymphocytes infiltration in CNS), GFAP (the glial fibrillar astrocytic protein, here used as a marker of astrocytosis) and IBA-1 (the ionized calcium binding adaptor molecule 1, here used as a marker of microglia activation) in cortical homogenates of young and old mice, and then to investigate whether, and to what extent, EAm administration could change these parameters.

**Figure 5:**
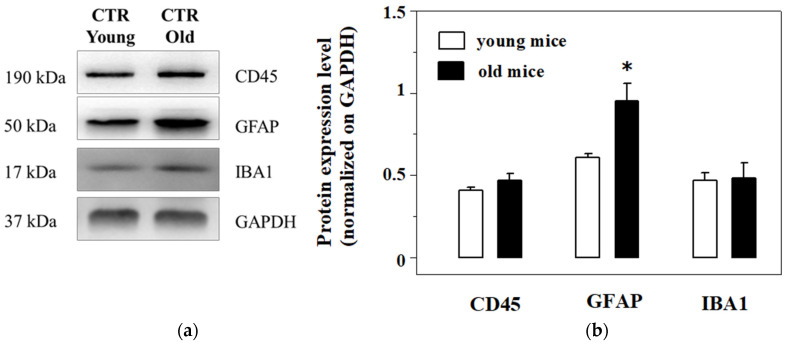
Western Blot analysis and quantification of the expression of the CD45, the glial fibrillar astrocytic protein (GFAP) and Ionized calcium binding adaptor molecule 1 (IBA-1) in cortical lysates from young and old mice. (**a**): representative blots of the GFAP, IBA-1 and CD45 immunostaining in cortical lysates from EAm-untreated young (empty bar) and old (gray bar) mice. The GAPDH protein was used as internal control. The blot is representative of five blots analyzing the lysates from 7 young and 6 old mice that were carried out on different days. (**b**): quantification of the change in CD45, GFAP and IBA-1 immunostaining in the cortical lysates from young (empty bar) and old (black bar) mice. Data represent the mean± S.E.M. Results are expressed as mean of the respective protein ÷ GAPDH ratio for the different animal groups. Statistical analysis was performed by applying ANOVA followed by Student t test analysis for direct comparison * *p* < 0.05 vs. respective control.

The Western blot analysis of the expression of the three proteins unveiled a significant increase of the GFAP expression in the cortical homogenates from old mice when compared to young, while CD45 and IBA1 were almost unmodified in aged mice cortical lysates when compared to the respective controls (Figure 5a,b).

**Figure 6:**
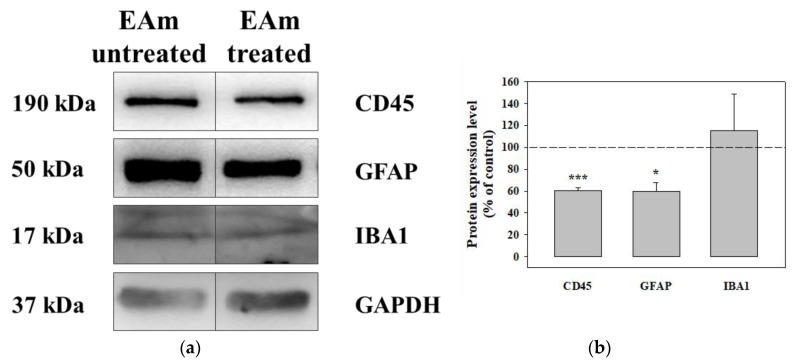

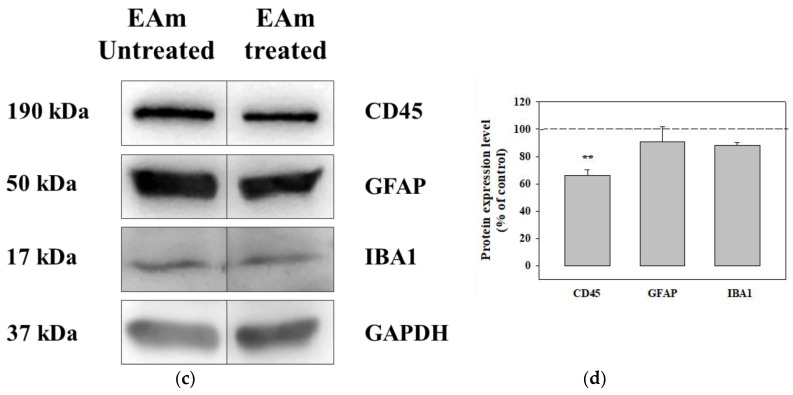
Effects of EAm treatment on the expression of the CD45, of the glial fibrillar astrocytic protein (GFAP), and of the ionized calcium binding adaptor molecule 1 (IBA-1), in cortical lysates from young and old mice. (**a**): representative blots of the GFAP, IBA-1 and CD45 immunostaining in cortical lysates from EAm-untreated and EAm-treated young mice. The GAPDH protein was used as internal control. (**b**): quantification of the change in CD45, GFAP and IBA-1 immunostaining in cortical lysates from EAm treated young mice, versus EAm untreated mice. Results are calculated as respective protein ÷ GAPDH ratio and are expressed as percentage of the EAm untreated mice. (**c**) representative blots of the GFAP, IBA-1 and CD45 immunostaining in cortical lysates from EAm-untreated and EAm-treated old mice. (**d**) as for (b), but for old mice. The blot is representative of five blots for young mice (seven EAm-untreated and seven EAm-treated young mice) and of five blots for old mice (eight EAm-untreated and 7 EAm-treated old mice) carried out in different days. Statistical analysis was performed by applying ANOVA followed by Student t test analysis for direct comparison * *p* < 0.05 vs. respective control; ** *p* < 0.01 vs. respective control; *** *p* < 0.001 vs. respective control.

The chronic administration of EAm caused a significant reduction of the CD45 protein content in both the EAm-treated young and old mice cortical homogenates (Figure 6a–c), when compared to untreated controls, as highlighted as changes with respect to controls (Figure 6b–d). EAm treatment also significantly reduced the expression of GFAP protein in cortical lysate from young mice (Figure 6a,b), but not in the old animals (Figure 6c,d), while IBA-1 was unmodified in both EAm-treated and untreated animal groups (Figure 6a–d).

### 2.6. Effects of the Oral Administration of EAm on the Cytokine Levels in the Cortex of Young and Aged Mice

**Figure 7:**
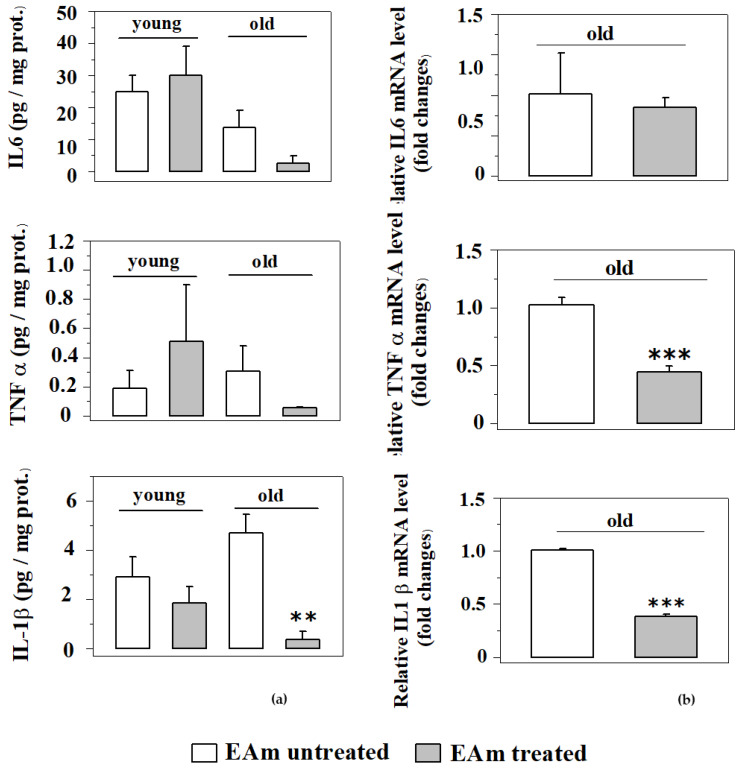
Effects of EAm treatment on the endogenous production of pro-inflammatory cytokines in the cortex of mice. The amount of the cytokines was measured as protein content (**a**) and as mRNA levels (**b**) in the respective mice (N = 4 animals/group). (**a**) Histograms show the mean values of protein expression (pg/mg) of TNF-α, IL-6 and IL-1β. Data are expressed as an average ± SEM (One Way ANOVA followed Tukey’s Multiple Comparison test ** *p* < 0.01 versus untreated mice). (**b**). Histograms show the mean values of mRNA levels of TNF-α, IL-6 and IL-1β. Data are expressed as mean ± SEM (Student’s t-test *** *p* < 0.001 versus old untreated mice).

Since EA is known to have, among others, anti-inflammatory [30] properties, we analyzed the impact of the EAm treatment on the production of selected pro-inflammatory cytokines (TNF-α, IL-1β and IL-6), either as protein level (measured with Elisa technique) or as mRNA expression (measured with quantitative PCR). As far as the cytokines levels are concerned, the endogenous amount of TNF-α and IL-1β proteins in the cortical lysates from the old mice does not significantly vary with respect to that detected in cortical lysates from young mice. The EAm administration failed to significantly affect the expression of the three cytokines in young mice, but significantly reduced the expression of IL-1β in EAm-treated old mice. Moreover, the amount of IL-6 and of TNF-α was slightly, although not significantly, modified in EAm-treated old mice when compared to respective control (Figure 7a). Since EAm-administration did not significantly modify the expression of the cytokines protein in EAm-untreated old mice when compared to the respective young mice, we limited the quantification of the mRNA to the old animals and quantified the impact of EAm on the mRNA content of the cytokines in the cortex of these mice. EAm-treatment significantly reduced the mRNA content relative to the TNF- and IL-1β proteins, leaving that of IL-6 unmodified.

## 3. Discussion

This work describes the results of a pilot study in mice, which aims to highlight EAm-induced effects that could support the use of this formulation for “nutraceutical” purposes.

The study focused on healthy old male mice in order, firstly, to recapitulate central functional impairments that typify aging and, secondly, to investigate whether EAm could recover some of the age-related derangements, being beneficial to brain functions.

As far as the first point is concerned, the results described confirm the development of functional impairments at noradrenergic nerve endings in the cortex of aged mice. The efficiency of NA exocytosis in the cortex is significantly reduced in old mice when compared to young ones, as already reported in a recent study (six and references therein), while its spontaneous release and uptake are not.

Concomitant with the noradrenergic impairment, the expression of GFAP in the cortex is largely increased, suggesting the occurrence of an age-dependent astrocytosis, at least in this region. To note, pathological alterations are restricted to astrocytes, since the activation of microglia (measured as increased IBA-1 expression) as well as lymphocytes infiltration (here measured as increased CD45+ positivity) were not observed.

These findings require attention, since it is widely recognized that the IS and the CNS message one to each other in a physiological interaction aimed at preserving central homeostasis: any condition that alters this physiological equilibrium favors central derangements.

Glial cells (the immunocompetent component of the CNS) consist of the resident microglia cells, the astrocytes and the infiltrating lymphocytes. In healthy condition, microglia are in the resting state, but, upon stimulation by circulating lymphocytes and/or local insults, it turns to the active state and mediate astrocytosis. Astrocytes, which are the most abundant central glial cells, are neuroprotective in healthy conditions and control the chemical transmission by finely tuning the bioavailability of the endogenous transmitters (particularly glutamate) in the synaptic cleft. When activated, astrocytes become hypertrophic (as indicated by the over-expression of GFAP) and lose their ability to control the biophase, but release cytokines, accelerating central pathological derangements [31,32,33].

It is proposed that astrocytosis is initiated by two main mechanisms: (i) the release of damage-associated molecules from activated microglia cells and circulating lymphocytes (i.e., the cytokines); (ii) the removal of inhibitory neuronal signaling(s) [34]. The first mechanism seems to not play a main role in aging, since increased infiltration of CD45+ cells and microglia activation were not evident in the cortex of aged mice when compared to young ones. Furthermore, a clear overproduction of pro-inflammatory cytokines was not detected (only a slight non-significant increase of TNF α and IL-6 was observed in the cortex of aged mice), compatible with the conclusion that microglia-mediated deleterious signaling, if present, was insufficient to trigger astrocytosis. Contrastingly, as far as the second mechanism is concerned, the concomitance of the reduced NA exocytosis and the GFAP overexpression could suggest a causative link between the two events. The hypothesis is supported by the notion that NA dictates the interaction between the IS and the CNS [35]. Actually, the amine hampers the release of cytokine by acting at noradrenergic receptors located in microglia and astrocytes, slowing the adoption of the inflammatory phenotype in these cells [36].

We propose that the reduced efficiency of NA exocytosis observed in aged brain might support neuroinflammation. As far as the noradrenergic transmission is concerned, it is worth remembering that noradrenergic nerve terminals do not make synaptic contact, but that the amine reaches by volume diffusion its receptors located postsynaptically [26,37,38]. The efficiency of the monoaminergic signaling therefore strictly depends on the amount of the amine that is released upon application of a depolarizing stimulus. Based on our observations, in the aged brain, the probability that NA can reach by non-synaptic volume diffusion its receptors on neighboring cells/terminals is largely diminished, minimizing the relevance of the noradrenergic innervation in the maintenance of the synaptic homeostasis, but concomitantly favoring the pathological activation of astrocytes and microglia.

Our results therefore suggest that (i) during aging the physiological tuning, linking astrocytes and neurons in the brain are impaired; (ii) noradrenergic defects negatively reverberate on the neuron-glia cross-talk, favoring astrocytosis and the overproduction of proinflammatory cytokines that in turn reverberate on nerve endings, worsening the deregulation of the noradrenergic innervation [39].

As far as the second aim of the study is concerned, we aimed at evaluating whether EAm orally administered for fourteen days dissolved in the drinking water could be beneficial to aging.

A large body of evidence indicates that pomegranate juice (a beverage particularly rich in EA and ETs) is beneficial in aged subjects [40,41,42], but the mechanism(s) is(are) far from being elucidated. In this context, our results from “ex-vivo, in vitro” experiments concerning the impact of EAm on NA release might be relevant to improve the comprehension of the molecular events that might account for the health effects elicited by pomegranate in elders.

The chronic administration of EAm did not cause persistent changes to the NA exocytosis from cortical nerve endings of young mice, possibly because this parameter is not impaired in these animals. However, EAm significantly potentiated the release of the amine from cortical synaptosomes of old mice. Again, the effect was specific to the mechanism of transmitter exocytosis, since the basal release, as well as the uptake of the amine, were not modified.

Based on the well-known anti-inflammatory and immunomodulatory activity of EA [13,43], we extended our studies to central inflammation and astrocytosis. The facilitation of NA exocytosis observed in EAm-treated old mice was paralleled by a significant reduction of the TNF-alpha and IL1beta mRNA contents and of the IL1beta protein level in this brain region. The data available are so far preliminary and deserve further investigation to be confirmed; nonetheless, the results are consistent with the proposed role of the NA-astroglia axis in the CNS during aging. We speculated that the EAm-induced restauration of NA exocytosis at noradrenergic nerve terminals could have favored the diffusion of the amine in the synaptic cleft, strengthening the aminergic control on neighboring astrocytes [37] and hampering the cytokine production in these cells. The observation that the GFAP expression in cortical homogenates from EAm treated old mice was still comparable to that observed in untreated animals disagrees with this conclusion, rather suggesting that EAm cannot revert the pathological phenotype of astrocytes.

An alternative hypothesis is that the central cytokines are released by circulating lymphocytes, which infiltrate the brain and exert constant immune surveillance on central function(s). Interestingly, although CD45 immunopositivity in the cortex is not significantly modified during aging, EAm administration significantly reduced the cortical CD45 signal in both the young and the old mice cortex, consistent with the conclusion that EAm could temporally break the “peripheral to central” IS-CNS cross-talk. The finding is particularly intriguing when considering that the migration of CD45+ lymphocytes from the periphery to the CNS is a mechanism involved in the onset and development of several immune diseases, including the autoimmune ones (i.e., the multiple sclerosis, [44,45]). This observation would support the use of EAm or derivatives for nutraceutical supplementation for the therapy of human subjects suffering from immune and autoimmune central diseases, as indeed already suggested in the literature [13].

Last but not least, it has been proposed that most of the “ex-vivo, in vitro” effects elicited by the EAm administration could be ascribed directly or indirectly to the antioxidant activities of EA. Notably, both CNS inflammation and altered central catecholamine transmission in part rely on oxidative stress that could be prevented by EA administration [46,47]. Urolithin A, which represents the main metabolite of EA, has anti-inflammatory properties and reduces the endogenous production of cytokines in inflammatory disease [48]. Furthermore, by controlling the redox equilibrium both in the periphery and in the CNS, EA and its main metabolites control the nuclear factor kappa B (NF-ĸB)-mediated pathways, then controlling inflammation and activation of lymphocytes as well as cytokines production [49]. Finally, EA also tunes the monoamino-oxidase activity, preserving endogenous catecholamines by their main metabolism, both peripherally and in the CNS. The possibility that the restoration of the NA transmission and the anti-inflammatory activity of EAm relies on its antioxidant activity is therefore attractive and surely deserves further studies to be correctly addressed.

Before drawing any conclusions, the behavioral studies deserve a brief comment. The reduced noradrenergic transmission in the cortex of the aged animals would be expected to favor the onset of an anxious phenotype. Differently from expectations, however, the univariate analysis of the results from the behavioral studies neither highlighted evidentiated clear changes in the spontaneous motor activity and in the curiosity in the aged mice, nor did it unveil any beneficial effects due to the EAm treatment. Differences in behavioral performances in the EAm-treated mice when compared to the untreated ones, however, were highlighted with the PCA analysis and could argue for a healthy effect of the treatment. Although the limited number of animals dedicated to the behavioral studies might have affected the analysis, the conclusion from both the univariate and the multivariate analysis appear to be consistent with the thesis, that the administration of “nutraceuticals” (administered both as food/beverage or concentrated in a dietary supplements) preferentially elicits a “trend” (often reported as “healthy effect”), instead of a clear improvement of behavioral skill(s), that is usually observed following the administration of therapeutics. Basically, although the “in vivo” data were largely preliminary, being restricted to one behavioral test and involving a limited number of animals, it seems conceivable to conclude that the “in vivo” analysis of the behavioral skills would not represent a suitable approach to highlight EAm-induced effects, that differently clearly emerge in “ex-vivo, in vitro” studies.

To conclude, the results described in this study improve our knowledge of the central cortical alterations that occur during aging, further highlighting the main role of the NA-glia axis in the development of the synaptic defects that typify aging. Furthermore, they unveil that the new formulation of EA exerts beneficial effects in the CNS of aged mice, posing the basis of its use as a new potential ingredient in functional food for elders. In particular, we here provide evidence that the chronic oral administration of EAm in old mice recovers the impaired NA exocytosis, but also reduces the production of proinflammatory cytokines. Furthermore, the EAm treatment also diminishes the population of CD45+ cells in the cortex, independently on the age of the animals, an effect that could support the use of EAm for the cure of central neuro-immunological diseases.

The question arises on the pharmacokinetic profile of the formulation, in terms of concentration of EA and/or UROs in the biological fluids and/or in the CNS in the EAm-treated mice. It is worth remembering that the microdispersion used in this work largely improves the solubility of EA in water, then favoring its intestinal absorption and distribution. Furthermore, pectin used as excipients would have positively affected the muco-adhesion, even influencing the gut-microbiota composition. Since EA is rapidly metabolized to urolithins (UROs), which are much better absorbed than EA and cross the blood brain barrier, the possibility exists that these metabolites, and not EA, could be responsible for most of the health effects here described. The reciprocal role of EA and URO in the effects elicited by EAm was not tackled so far, since our priority was to emphasize whether the EAm could have clear and quantifiable beneficial effects in aged mice. Nonetheless, although insufficient to postulate the exact mechanism of action, the results of this first study could be consistent with both peripheral and central events and might involve either EA or UROs-mediated effects.

We are perfectly aware that further studies are needed to better define the pharmacokinetic and the pharmacological profiles of the EAm-induced effects in aged mice, as well as to strengthen the present findings. However, despite these limitations, we are convinced that our observations further pose the basis of the use of EAm as a “nutraceutical” intervention to healthy aging.

## 4. Materials and Methods 

### 4.1. EA Microdispersion and EA Drug Loading Analysis of the Drinking Daily Solutions

EA microdispersion (EAm), using pectin as a food compatible polymeric matrix, was realized by a BUCHI B-290 mini-dryer (Flawil, Switzerland), as previously reported by the authors [20]. EAm was employed to prepare the drinking daily solutions for the animal treatment dissolving EAm in mineral water 1:1 *w*/*w*. The EA drug loading (DL) % (*w*/*v*) of EAm was previously checked by HPLC [20] and it was spectrophotometrically monitored in the daily drinking solutions. All the UV-visible spectra (210–450 nm) were recorded by an Agilent 8453 spectrophotometer with 1 nm resolution (Waldbronn, Germany), using rectangular quartz cuvettes with 1 cm path length. The total spectrum of each formulation, opportunely diluted in methanol (1:50), was collected at room temperature in duplicate, against methanol as blank, and the results were averaged. Standard normal variate (SNV) [23], as a preprocessing technique with the aim to remove light scattering or other interfering phenomena, was applied. The EA DL % of the drinking daily solutions was determined comparing the absorbance value at ʎ = 255 nm, with a calibration curve of pure EA in methanol (R^2^ = 0.9958) previously built.

### 4.2. Animals

Mice (male, strain C57BL/6J) were obtained from Charles River (Calco, Italy) and were housed in the animal facility of the Department of Pharmacy, Section of Pharmacology and Toxicology, School of Medical and Pharmaceutical Sciences, University of Genoa (authorization n. 484 of 2004, June, 8th). Young (three months old) and old (twenty months old) mice were killed by cervical dislocation and immediately decapitated to collect the cortices. The experimental procedures were in accordance with the European legislation (Directive 2010/63/EU for animal experiments), the ARRIVE guidelines and the 8th Edition of the “Guide for the care and Use in laboratory-animals”, and they were approved by the Animal Subjects Review Board of the University of Genoa and by the Italian Ministry of Health (DDL 26/2014 and previous legislation; date of approval June, 2015, number of approval 02/10/06/2015-OPBA).

### 4.3. Animal Treatment

Ellagic acid (EA) was obtained by Sigma Aldrich (Milan, Italy). Mice were randomly assigned to the following groups: untreated young mice; EA-treated young mice; untreated old mice; EA-treated old mice. The number of animals in each group was defined on the basis of previous studies [6], since preliminary data allowing one to define the effect size and the standard deviation were not available in literature. EAm was given orally, dissolved in the drinking water [22,50,51]. After a three-day trial to determine the amount of water consumed by each group of mice, mice were treated with EAm (50 mg/Kg/day) for 14 days. The drug dose was selected based on data from the literature [52,53,54]. Animals were checked daily for the EAm intake and the weight was controlled before (day 0), during (day 7), and at the end (day 14) of the EAm administration.

### 4.4. Behavioural Tests

The light-dark box was used to test unconditioned anxiety and exploratory behavior of mice. The text is based on the innate aversion of mice to bright light in novel areas. The apparatus consists of a box divided by a connecting door (3 × 2 cm, length × height), into a light chamber (35 cm × 30 cm × 21 cm, length × width × height) and a dark chamber (35 cm × 30 cm × 21 cm, length × width × height). The light chamber is entirely painted white. Mice are placed in the center of the light chamber and behavior is monitored for 5 min via a micro-camera. The anxiety parameters were defined according to previous studies as: (i) the number of transitions between the two chambers and (ii) the time spent in the light side of the maze. The time spent in the light chamber was expressed in seconds [55]. Animals behavioral skills were checked before (day 0), during (day 7) and at the end (day 14) of the EA administration.

### 4.5. Preparation of Synaptosomes

Purified synaptosomes were prepared as already described in the literature [56]. The synaptosomal pellets were resuspended in a physiological solution with the following composition (mM): NaCl, 140; KCl, 3; MgSO_4_, 1.2; CaCl_2_, 1.2; NaH_2_PO_4_, 1.2; NaHCO_3_, 5; HEPES, 10; glucose, 10; pH 7.2–7.4.

### 4.6. Experiments of Release

Synaptosomes were isolated from the cortices of the mice belonging to the following four groups: untreated young mice; EA-treated young mice; untreated old mice; EA-treated old mice. Synaptosomes were incubated for 15 min a 37 °C with [^3^H]noradrenaline ([^3^H]NA; f.c.: 30 nM) in the presence of 0.1 µM 6-nitroquipazine and 0.1 µM GBR12909, to avoid false labelling of serotonergic and dopaminergic terminals, respectively [51]. Identical portions of the synaptosomal suspensions were layered on microporous filters at the bottom of parallel chambers in a Superfusion System (Ugo Basile, Comerio, Varese, Italy; [57]), and maintained at 37 °C.

Synaptosomes isolated from the mice of the four experimental animal groups analyzed for behavioral skills (i.e., the untreated young mice, the EAm-treated young mice, the untreated old mice, the EAm-treated old mice) labelled with [^3^H]noradrenaline ([^3^H]NA), to monitor the efficiency of NA, uptake and release were analyzed for their release efficiency. The NA uptake in the cortical synaptosomes was quantified as the amount of tritium in the synaptosomal fraction. The basal release of the radioactive tracer was quantified as the amount of tritium released in the 3 min fraction collected immediately before that containing the depolarization-evoked release of amine. Synaptosomes were then transiently (90 s) exposed, at t = 39 min, to high KCl containing medium (12 mM, NaCl substituting for an equimolar concentration of KCl), as already described in previous works [6,21,49,54]. The K^+^-induced overflow was expressed as “induced overflow (%)”, and it was quantified as difference between the percentage of radioactivity collected during and after the depolarization pulse (evoked release) and that into the fractions collected before and after the evoked outflow.

### 4.7. Cytokines Measurements

Tissue samples were obtained from different groups (young mice, old mice, ellagic acid treated mice) after 14 days treatment. Cytokines (IL-1β, IL-6 and TNF-α) production were quantify by ELISA quantikine kit (R&D system), according to the manufacturer’s instructions [58]. The cytokine expression was normalized against the total protein content for a given sample (mg of protein), as measured using the bicinchoninic acid (BCA) assay (Thermo Scientific, Rockford, IL, USA).

### 4.8. qRT-PCR Cytokine mRNA Quantification

Total RNA (1 μg) was reverse transcribed into first-strand cDNA by using SuperScript^®^ VILO™ cDNA Synthesis Kit (according to the manufacturer’s instructions), in a final volume of 20 μL. HPRT-1 was used as the reference housekeeping gene in RT-PCR assays. Amplification of inflammatory, antioxidant target genes and HPRT-1 was conducted with 50 ng of cDNA in 20 μL of the reaction mixture by gene-specific primers, using fluorogenic probes (TaqMan) and TaqMan^®^ Universal PCR Master Mix, No AmpErase^®^ UNG (Applied Biosystems). TaqMan primer/probes sets were used spanning exon-exon junctions, for mouse IL-1β (Mm00434228_m1), IL-6 (Mm00446190_m1), TNF-α (Mm00443259_g1) and the housekeeping gene HPRT-1 (Mm00446968_m1) were used in PCR reactions. PCR reactions were run in a 96-well format on ViiA™ 7 Real-Time PCR System (Applied Biosystems), using universal cycling conditions (95 °C, 10 min; 95 °C, 15 s; and 60 °C, 1 min for 40 cycles).

### 4.9. Western Blot Analysis

Mouse cortical lysates were prepared as already described [55,57] and then separated by SDS-10% PAGE (40 µg/lane) and blotted onto PVDF membrane. Membranes were probed with the following primary antibodies overnight at 4 °C: rabbit anti-CD45 (1:2000, Cell Signaling Technology, USA), mouse anti-GFAP (1:5000, Sigma, USA), rabbit anti-IBA1 (1:1000, FUJIFILM, Japan), and 1h at room temperature with rabbit anti-GAPDH (1:5000, Cell Signaling Technology, USA), and after extensive washes in t-TBS, were incubated for 1 h at room temperature with the appropriate horseradish peroxidase-linked secondary antibodies (1:20000). Immunoblots were visualized using an ECL (enhanced chemiluminescence) Western blotting detection system. Images were acquired using the Alliance LD6 images capture system (Uvitec, Cambridge, UK) and analyzed by UVI-1D software (Uvitec, Cambridge, UK).

### 4.10. Calculations and Statistical Analysis

The univariate statistical analysis was carried out by using the KyPlot program. An analysis of variance was performed by ANOVA followed by Tukey’s multiple comparison test; direct comparisons were performed by Student’s t-test. Data were considered significant for *p* < 0.05 at least. The multivariate statistical analysis was performed using the Chemometric Agile Tool (CAT) software(ITALY) freely downloadable from http://gruppochemiometria.it/index.php/software [59].

### 4.11. Drugs

Notably, 1-[7,8 3H]-noradrenaline (specific activity 39 Ci mmol-1), was from Amersham Radiochemical Centre (Buckinghamshire, UK). The ellagic acid was from Sigma Aldrich (Mi., Italy). 6-Nitroquipazine maleate was donated from Duphar, Amsterdam, the Netherlands. 1-(2-(Bis-(4-fluorophenyl) methoxy) ethyl)—4-(3-phenylpropyl) piperazine dihydrochloride (GBR12909) was purchased from Tocris Bioscience (Bristol, UK). Anti-mouse and anti-rabbit secondary antibodies were from Sigma-Aldrich (USA). The SuperScript^®^ VILO™ cDNA Synthesis Kit was from Life Technologies (USA).

This could account for the effects here described, allowing the conclusion that either EA or UROs could have a role in determining their onset.

## Abbreviations

EA	Ellagic Acid
EAm	Ellagic Acid Micro-Dispersion
CNS	Central Nervous System
ETs	Ellagitannins
UROs	Urolithins
IS	Immune System
DL	Drug Loading
SNV	Standard Normal Variate
PCA	Principal Component Analysis
PC	Principal Component
GFAP	Glial Fibrillar Astrocytic Protein
[^3^H]NA	[^3^H]noradrenaline
IBA-1	Ionized Calcium Binding Adaptor Molecule
CD45	Classification Determinant 45
TNF-	Tumor Necrosis Factor-
IL-1β	Interleukin-1β
IL-6	Interleukin-6
PCR	Polymerase Chain Reaction
RT-PCR	Real Time-PCR
NA	Noradrenaline

## References

[1] Deak F. (2014). Neuronal Vesicular Trafficking and Release in Age-Related Cognitive Impairment. J. Gerontol. Ser. A Biol. Sci. Med Sci..

[2] Mather M., Harley C.W. (2016). The Locus Coeruleus: Essential for Maintaining Cognitive Function and the Aging Brain. Trends Cogn. Sci..

[3] Segovia G., Arco A., Mora F. (2009). Environmental Enrichment, Prefrontal Cortex, Stress, and Aging of the Brain. J. Neural Transm..

[4] Liguz-Lecznar M., Lehner M., Kaliszewska A., Zakrzewska R., Sobolewska A., Kossut M. (2015). Altered Glutamate/GABA Equilibrium in Aged Mice Cortex Influences Cortical Plasticity. Brain Struct. Funct..

[5] Pittaluga A. (2019). Acute Functional Adaptations in Isolated Presynaptic Terminals Unveil Synaptosomal Learning and Memory. Int. J. Mol. Sci..

[6] Bonfiglio T., Vergassola M., Olivero G., Pittaluga A. (2019). Environmental Training and Synaptic Functions in Young and Old Brain: A Presynaptic Perspective. Curr. Med. Chem..

[7] Feart C. (2019). Nutrition and Frailty: Current Knowledge. Prog. Neuro Psychopharmacol. Biol. Psychiatry.

[8] Ogle W.O., Speisman R.B., Ormerod B.K. (2013). Potential of Treating Age-Related Depression and Cognitive Decline with Nutraceutical Approaches: A Mini-Review. Gerontology.

[9] Schättin A., Baur K., Stutz J., Wolf P., de Bruin E.D. (2016). Effects of Physical Exercise Combined with Nutritional Supplements on Aging Brain Related Structures and Functions: A Systematic Review. Front. Aging Neurosci..

[10] Bigliardi B., Galati F. (2013). Innovation Trends in the Food Industry: The Case of Functional Foods. Trends Food Sci. Technol..

[11] Iwatani S., Yamamoto N. (2019). Functional Food Products in Japan: A Review. Food Sci. Hum. Wellness.

[12] Shahidi F., Ambigaipalan P. (2015). Phenolics and Polyphenolics in Foods, Beverages and Spices: Antioxidant Activity and Health Effects–A Review. J. Funct. Foods.

[13] Ahmed T.N., Setzer W., Fazel Nabavi S., Erdogan Orhan I., Braidy N., Sobarzo-Sanchez E., Mohammad Nabavi S. (2016). Insights into Effects of Ellagic Acid on the Nervous System: A Mini Review. Curr. Pharm. Des..

[14] Alfei S., Turrini F., Catena S., Zunin P., Grilli M., Pittaluga A.M., Boggia R. (2019). Ellagic Acid a Multi-Target Bioactive Compound for Drug Discovery in CNS? A Narrative Review. Eur. J. Med. Chem..

[15] Kim Y.E., Hwang C.J., Lee H.P., Kim C.S., Son D.J., Ham Y.W., Hellström M., Han S.-B., Kim H.S., Park E.K. (2017). Inhibitory Effect of Punicalagin on Lipopolysaccharide-Induced Neuroinflammation, Oxidative Stress and Memory Impairment via Inhibition of Nuclear Factor-KappaB. Neuropharmacology.

[16] Welcome M.O. (2020). Neuroinflammation in CNS Diseases: Molecular Mechanisms and the Therapeutic Potential of Plant Derived Bioactive Molecules. PharmaNutrition.

[17] Bala I., Bhardwaj V., Hariharan S., Kumar M.N.V.R. (2006). Analytical Methods for Assay of Ellagic Acid and Its Solubility Studies. J. Pharm. Biomed. Anal..

[18] Tomás-Barberán F.A., García-Villalba R., González-Sarrías A., Selma M.V., Espín J.C. (2014). Ellagic Acid Metabolism by Human Gut Microbiota: Consistent Observation of Three Urolithin Phenotypes in Intervention Trials, Independent of Food Source, Age, and Health Status. J. Agric. Food Chem..

[19] González-Sarrías A., García-Villalba R., Núñez-Sánchez M.Á., Tomé-Carneiro J., Zafrilla P., Mulero J., Tomás-Barberán F.A., Espín J.C. (2015). Identifying the Limits for Ellagic Acid Bioavailability: A Crossover Pharmacokinetic Study in Healthy Volunteers after Consumption of Pomegranate Extracts. J. Funct. Foods.

[20] Alfei S., Turrini F., Catena S., Zunin P., Parodi B., Zuccari G., Pittaluga A.M., Boggia R. (2019). Preparation of Ellagic Acid Micro and Nano Formulations with Amazingly Increased Water Solubility by Its Entrapment in Pectin or Non-PAMAM Dendrimers Suitable for Clinical Applications. New J. Chem..

[21] Turrini F., Boggia R., Donno D., Parodi B., Beccaro G., Baldassari S., Signorello M.G., Catena S., Alfei S., Zunin P. (2020). From Pomegranate Marcs to a Potential Bioactive Ingredient: A Recycling Proposal for Pomegranate-Squeezed Marcs. Eur. Food Res. Technol..

[22] Bonfiglio T., Olivero G., Merega E., Di Prisco S., Padolecchia C., Grilli M., Milanese M., Di Cesare Mannelli L., Ghelardini C., Bonanno G. (2017). Prophylactic versus Therapeutic Fingolimod: Restoration of Presynaptic Defects in Mice Suffering from Experimental Autoimmune Encephalomyelitis. PLoS ONE.

[23] Barnes R.J., Dhanoa M.S., Lister S.J. (1989). Standard Normal Variate Transformation and De-Trending of Near-Infrared Diffuse Reflectance Spectra. Appl. Spectrosc..

[24] Wold S., Esbensen K., Geladi P. (1987). Principal Component Analysis. Chemom. Intell. Lab. Syst..

[25] Jolliffe I.T. (2002). Principal Component Analysis.

[26] Pittaluga A. (2016). Presynaptic Release-Regulating MGlu1 Receptors in Central Nervous System. Front. Pharmacol..

[27] Rodríguez J.J., Yeh C.-Y., Terzieva S., Olabarria M., Kulijewicz-Nawrot M., Verkhratsky A. (2014). Complex and Region-Specific Changes in Astroglial Markers in the Aging Brain. Neurobiol. Aging.

[28] Ritzel R.M., Crapser J., Patel A.R., Verma R., Grenier J.M., Chauhan A., Jellison E.R., McCullough L.D. (2016). Age-Associated Resident Memory CD8 T Cells in the Central Nervous System Are Primed To Potentiate Inflammation after Ischemic Brain Injury. J. Immunol..

[29] Zöller T., Attaai A., Potru P., Ruß T., Spittau B. (2018). Aged Mouse Cortical Microglia Display an Activation Profile Suggesting Immunotolerogenic Functions. Int. J. Mol. Sci..

[30] BenSaad L.A., Kim K.H., Quah C.C., Kim W.R., Shahimi M. (2017). Anti-Inflammatory Potential of Ellagic Acid, Gallic Acid and Punicalagin A&B Isolated from Punica Granatum. BMC Complementary Altern. Med..

[31] Matias I., Morgado J., Gomes F.C.A. (2019). Astrocyte Heterogeneity: Impact to Brain Aging and Disease. Front. Aging Neurosci..

[32] Gambino C.M., Sasso B.L., Bivona G., Agnello L., Ciaccio M. (2019). Aging and Neuroinflammatory Disorders: New Biomarkers and Therapeutic Targets. Curr. Pharm. Des..

[33] Valles S.L., Iradi A., Aldasoro M., Vila J.M., Aldasoro C., Torre J., Campos-Campos J., Jorda A. (2019). Function of Glia in Aging and the Brain Diseases. Int. J. Med Sci..

[34] Selmeczy Z., Vizi E.S., Csóka B., Pacher P., Haskó G. (2008). Role of Nonsynaptic Communication in Regulating the Immune Response. Neurochem. Int..

[35] Garwood C.J., Ratcliffe L.E., Simpson J.E., Heath P.R., Ince P.G., Wharton S.B. (2017). Review: Astrocytes in Alzheimer’s Disease and Other Age-Associated Dementias: A Supporting Player with a Central Role. Neuropathol. Appl. Neurobiol..

[36] Zepeda R., Contreras V., Pissani C., Stack K., Vargas M., Owen G.I., Lazo O.M., Bronfman F.C. (2016). Venlafaxine Treatment after Endothelin-1-Induced Cortical Stroke Modulates Growth Factor Expression and Reduces Tissue Damage in Rats. Neuropharmacology.

[37] Vizi E.S., Kiss J.P., Lendvai B. (2004). Nonsynaptic Communication in the Central Nervous System. Neurochem. Int..

[38] Pittaluga A. (2017). CCL5–Glutamate Cross-Talk in Astrocyte-Neuron Communication in Multiple Sclerosis. Front. Immunol..

[39] Bharani K.L., Derex R., Granholm A.-C., Ledreux A. (2017). A Noradrenergic Lesion Aggravates the Effects of Systemic Inflammation on the Hippocampus of Aged Rats. PLoS ONE.

[40] Siddarth P., Li Z., Miller K.J., Ercoli L.M., Merril D.A., Henning S.M., Heber D., Small G.W. (2019). Randomized Placebo-Controlled Study of the Memory Effects of Pomegranate Juice in Middle-Aged and Older Adults. Am. J. Clin. Nutr..

[41] Bookheimer S.Y., Renner B.A., Ekstrom A., Li Z., Henning S.M., Brown J.A., Jones M., Moody T., Small G.W. (2013). Pomegranate Juice Augments Memory and FMRI Activity in Middle-Aged and Older Adults with Mild Memory Complaints. Evid. Based Complementary Altern. Med..

[42] Ropacki S.A., Patel S.M., Hartman R.E. (2013). Pomegranate Supplementation Protects against Memory Dysfunction after Heart Surgery: A Pilot Study. Evid. Based Complementary Altern. Med..

[43] Danesi F., Ferguson L. (2017). Could Pomegranate Juice Help in the Control of Inflammatory Diseases?. Nutrients.

[44] Sanadgol N., Golab F., Tashakkor Z., Taki N., Moradi Kouchi S., Mostafaie A., Mehdizadeh M., Abdollahi M., Taghizadeh G., Sharifzadeh M. (2017). Neuroprotective Effects of Ellagic Acid on Cuprizone-Induced Acute Demyelination through Limitation of Microgliosis, Adjustment of CXCL12/IL-17/IL-11 Axis and Restriction of Mature Oligodendrocytes Apoptosis. Pharm. Biol..

[45] Busto R., Serna J., Perianes-Cachero A., Quintana-Portillo R., García-Seisdedos D., Canfrán-Duque A., Paino C.L., Lerma M., Casado M.E., Martín-Hidalgo A. (2018). Ellagic Acid Protects from Myelin-Associated Sphingolipid Loss in Experimental Autoimmune Encephalomyelitis. Biochim. Biophys. Acta BBA Mol. Cell Biol. Lipids.

[46] Farbood Y., Sarkaki A., Dianat M., Khodadadi A., Haddad M.K., Mashhadizadeh S. (2015). Ellagic Acid Prevents Cognitive and Hippocampal Long-Term Potentiation Deficits and Brain Inflammation in Rat with Traumatic Brain Injury. Life Sci..

[47] Baluchnejadmojarad T., Rabiee N., Zabihnejad S., Roghani M. (2017). Ellagic Acid Exerts Protective Effect in Intrastriatal 6-Hydroxydopamine Rat Model of Parkinson’s Disease: Possible Involvement of ERβ/Nrf2/HO-1 Signaling. Brain Res..

[48] Fu X., Gong L.-F., Wu Y.-F., Lin Z., Jiang B.-J., Wu L., Yu K.-H. (2019). Urolithin A Targets the PI3K/Akt/NF-ΚB Pathways and Prevents IL-1β-Induced Inflammatory Response in Human Osteoarthritis: In Vitro and in Vivo Studies. Food Funct..

[49] Ebrahimi R., Sepand M.R., Seyednejad S.A., Omidi A., Akbariani M., Gholami M., Sabzevari O. (2019). Ellagic Acid Reduces Methotrexate-Induced Apoptosis and Mitochondrial Dysfunction via up-Regulating Nrf2 Expression and Inhibiting the IĸBα/NFĸB in Rats. DARU J. Pharm. Sci..

[50] Di Prisco S., Summa M., Chellakudam V., Rossi P.I.A., Pittaluga A. (2012). RANTES-Mediated Control of Excitatory Amino Acid Release in Mouse Spinal Cord: RANTES Modulates Glutamate Transmission. J. Neurochem..

[51] Pittaluga A., Raiteri L., Longordo F., Luccini E., Barbiero V.S., Racagni G., Popoli M., Raiteri M. (2007). Antidepressant Treatments and Function of Glutamate Ionotropic Receptors Mediating Amine Release in Hippocampus. Neuropharmacology.

[52] Girish C., Raj V., Arya J., Balakrishnan S. (2012). Evidence for the Involvement of the Monoaminergic System, but Not the Opioid System in the Antidepressant-like Activity of Ellagic Acid in Mice. Eur. J. Pharmacol..

[53] Dhingra D., Chhillar R. (2012). Antidepressant-like Activity of Ellagic Acid in Unstressed and Acute Immobilization-Induced Stressed Mice. Pharmacol. Rep..

[54] Lorigooini Z., Salimi N., Soltani A., Amini-Khoei H. (2019). Implication of NMDA-NO Pathway in the Antidepressant-like Effect of Ellagic Acid in Male Mice. Neuropeptides.

[55] Bonfiglio T., Olivero G., Vergassola M., Di Cesare Mannelli L., Pacini A., Iannuzzi F., Summa M., Bertorelli R., Feligioni M., Ghelardini C. (2019). Environmental Training Is Beneficial to Clinical Symptoms and Cortical Presynaptic Defects in Mice Suffering from Experimental Autoimmune Encephalomyelitis. Neuropharmacology.

[56] Mairesse J., Gatta E., Reynaert M.-L., Marrocco J., Morley-Fletcher S., Soichot M., Deruyter L., Camp G.V., Bouwalerh H., Fagioli F. (2015). Activation of Presynaptic Oxytocin Receptors Enhances Glutamate Release in the Ventral Hippocampus of Prenatally Restraint Stressed Rats. Psychoneuroendocrinology.

[57] Zucchini S., Pittaluga A., Brocca-Cofano E., Summa M., Fabris M., De Michele R., Bonaccorsi A., Busatto G., Barbanti-Brodano G., Altavilla G. (2013). Increased Excitability in Tat-Transgenic Mice: Role of Tat in HIV-Related Neurological Disorders. Neurobiol. Dis..

[58] Romano I., Ayadi F., Rizzello L., Summa M., Bertorelli R., Pompa P.P., Brandi F., Bayer I.S., Athanassiou A. (2015). Controlled Antiseptic/Eosin Release from Chitosan-Based Hydrogel Modified Fibrous Substrates. Carbohydr. Polym..

[59] Leardi R., Melzi C., Polotti G. Chemometric Agile Software (CAT). http://gruppochemiometria.it/index.php/software.

